# Current treatment outcomes and care pathways for people with comorbid physical and mental health conditions using NHS Talking Therapies services in the UK: systematic review of quantitative studies

**DOI:** 10.1192/bjo.2025.10957

**Published:** 2026-02-05

**Authors:** Mary C. Abichi, Alice Davis, Hannah Proudfoot, Sam Norton, Rona Moss-Morris, Joanna Hudson

**Affiliations:** Department of Health Psychology, King’s College London, Guy’s Hospital, London, UK; Department of Psychology, King’s College London, London, UK; Mayden, Bath, UK; Department of Psychological Medicine, Institute of Psychiatry, Psychology & Neuroscience, King’s College London, London, UK

**Keywords:** Chronic conditions, depressive disorders, anxiety- or fear-related disorders, comorbidity

## Abstract

**Background:**

In 2018, the UK government commissioned National Health Service Talking Therapies (NHS TT) services to provide integrated mental and physical health care for individuals with a long-term condition (LTC) and coexisting depression and/or anxiety. Nevertheless, evidence on the effectiveness of NHS TT in physical LTCs remains inconsistent.

**Aims:**

This review aims to evaluate the impact of NHS TT on mental health outcomes among adults with physical LTCs.

**Method:**

We conducted a systematic review and meta-analysis of quantitative studies published between 2008 and 2024. We used several databases for the search, including Embase, MEDLINE, Cochrane Library, NHS Evidence, PsycINFO, Bielefeld Academic Search Engine and ProQuest. We combined terms related to NHS TT, LTCs and mental health outcomes to identify eligible studies. The Population, Intervention, Comparison, Outcomes and Study framework guided the development of the inclusion criteria. We employed the random-effects model for meta-analysis and assessed heterogeneity bias using the *I* ^2^ statistic, and the Newcastle–Ottawa scale to evaluate the overall quality of the evidence.

**Results:**

Twenty-four studies met the inclusion criteria. The meta-analysis revealed a significant pre–post NHS TT intervention effect on reliable improvement (odds ratio 0.77, 95% CI: 0.60–0.98) and reliable recovery (odds ratio 0.80, CI: 0.68–0.95). There were no significant differences in NHS TT accessibility (e.g. treatment engagement) between participants with and without LTCs (odds ratio 0.97, 95% CI: 0.82–1.14). However, heterogeneity between the studies was high (>90%).

**Conclusions:**

The observed evidence provides reassurance for individuals with LTCs engaging with treatment; however, the association with post-treatment distress is still of concern. Furthermore, extensive and rigorous research is needed to strengthen and guide service development for individuals with LTCs, thereby improving effectiveness.

In England, an estimated 15 million people (31% of the adult population) live with one or more physical long-term conditions (LTCs), and around 30% of these individuals also experience co-occurring symptoms of depression or anxiety.^1^ This combination is conducive to a poorer quality of life, reduced ability to self-manage physical LTCs, increased healthcare utilisation and worse clinical outcomes, including a greater risk of mental illness relapse or physical symptom exacerbation.^2–4^ According to the King’s Fund, the National Health Service (NHS) spends between £8 billion and £13 billion annually on caring for people with coexisting mental and physical health conditions.^1,5,6^

NHS Talking Therapies (NHS TT), previously known as Improving Access to Psychological Therapies (IAPT), is a large-scale, publicly funded programme designed to provide evidence-based psychological interventions for common mental health conditions such as depression and anxiety disorders.^7^ The service predominantly offers cognitive–behavioural therapy (CBT) and related evidence-based modalities, including behavioural activation, interpersonal therapy, counselling for depression and guided self-help, delivered with the National Institute for Health Care Excellence (NICE)-recommended step-care model. Long-term psychodynamic psychotherapy and specialist secondary care psychological treatments fall outside the remit of NHS TT. The programme aims to increase access to timely, structured, outcome-focused therapy for those of working age, with its expansion to include those with complex needs and comorbidities. With the recent expansion and outcome-focused approach, transparency around outcomes for vulnerable groups, such as those with LTCs, has been lacking, as the one-size-fits-all (stepped-care) treatment model remains in place.

In response to the rising burden of having one or more LTCs within NHS TT, the Five-Year Forward View for Mental Health policy has called for the development of integrated models of care for people living with both physical LTCs and mental health conditions.^8^ This policy was underpinned by evidence from randomised controlled trials (RCTs) that support the effectiveness of integrated mental and physical healthcare approaches in improving mental health in this population.^9–11^ To support the implementation of this policy, in 2018 NHS TT services expanded services to offer LTC-tailored interventions (NHS TT-LTC) as an integrated care pathway to close the gap between those with LTCs and those without.^12^ Despite this strategic shift, the effectiveness of NHS TT-LTC services for individuals with LTCs remains poorly examined.

This systematic review and meta-analysis aimed to address this gap by evaluating the effectiveness of NHS TT services in treating depression and anxiety in adults with LTCs. A secondary aim was to synthesise evidence on variability in accessibility, engagement and outcomes within NHS TT services for LTC populations, including the potential moderators of treatment effects.

## Method

The systematic review and meta-analysis was conducted and reported in accordance with the Preferred Reporting Items for Systematic Reviews and Meta-Analyses (PRISMA) guidelines, and was registered in the International Prospective Register of Systematic Reviews (PROSPERO) under ID: CRD42022379786. A completed PRISMA checklist is available in the supplementary material available at https://doi.org/10.1192/bjo.2025.10957.

### Literature search

We conducted a systematic literature search on 10 November 2022, and updated it using the same search strategy on 31 December 2024. The initial search terms were informed by a prior systematic review,^13^ using broad descriptions to capture the service IAPT. The updated search strategy included two additional terms, ‘NHS Talking Therapies’ or ‘NHS TT’, to reflect the recent rebranding of the service. To maximise sensitivity, we did not restrict the search using terms such as ‘long-term conditions’, ‘LTCs’ or ‘comorbidities’, because these are often inconsistently reported in titles and abstracts. Persistent physical symptoms (PPS) were considered within the scope of LTCs for this review. Searches were conducted in the NHS Evidence Healthcare database, which integrates Embase, MEDLINE, the Cochrane Library, PsychINFO and grey literature sources, including BASE, ProQuest and EThOS. In addition, we performed manual backwards-and-forwards citation tracking of included studies to identify further eligible studies.

### Eligibility criteria

We used the same inclusion criteria reported in the Prospero Protocol: https://www.crd.york.ac.uk/prospero/display_record.php?ID=CRD42022379786

### Inclusion criteria

Studies were included if they:

- reported real-world data from NHS TT service;
- used the self-reported outcomes Patient Health Questionnaire 9 (PHQ-9 Depression) and/or General Anxiety Disorder 7 (GAD-7) Anxiety scale;
- reported data in either binary format (e.g. reliable recovery, reliable improvement) or continuous format. Reliable recovery is defined as… Reliable Improvement is defined as… Reliable deterioration is defined as (NHS Digital 2018);
- provided data comparing LTC populations with non-LTC;
- provided data comparing tailored NHS TT interventions for LTC populations with non-tailored core NHS TT interventions for individuals with LTCs, and/or looked at the size of treatment effects using pre–post change scores for an LTC-adapted intervention. For this review, an LTC is defined as a chronic physical condition that cannot be cured. This includes, but is not limited to, diabetes, rheumatoid arthritis, chronic obstructive pulmonary disease, coronary heart disease and PPS (defined as disease-specific symptoms that organic pathology cannot explain,^14^ also known as medically unexplained symptoms in previous literature). A tailored LTC intervention is defined as a CBT intervention whose description specifically references changes in the application of CBT therapeutic components in the context of living with LTCs;
- included the term disability, defined under the Disability Discrimination Act as ‘someone who has a physical or mental impairment that has a substantial and long-term adverse effect on his or her ability to carry out normal day-to-day activities’.

### Exclusion criteria

Studies that were excluded:

- protocol papers and conference abstracts;
- reported self-report scales of depression and anxiety that were not PHQ-9 or GAD-7, including the following;
- case studies and qualitative papers that involved healthcare professionals as the sample.

### Study selection

**Fig. 1:**
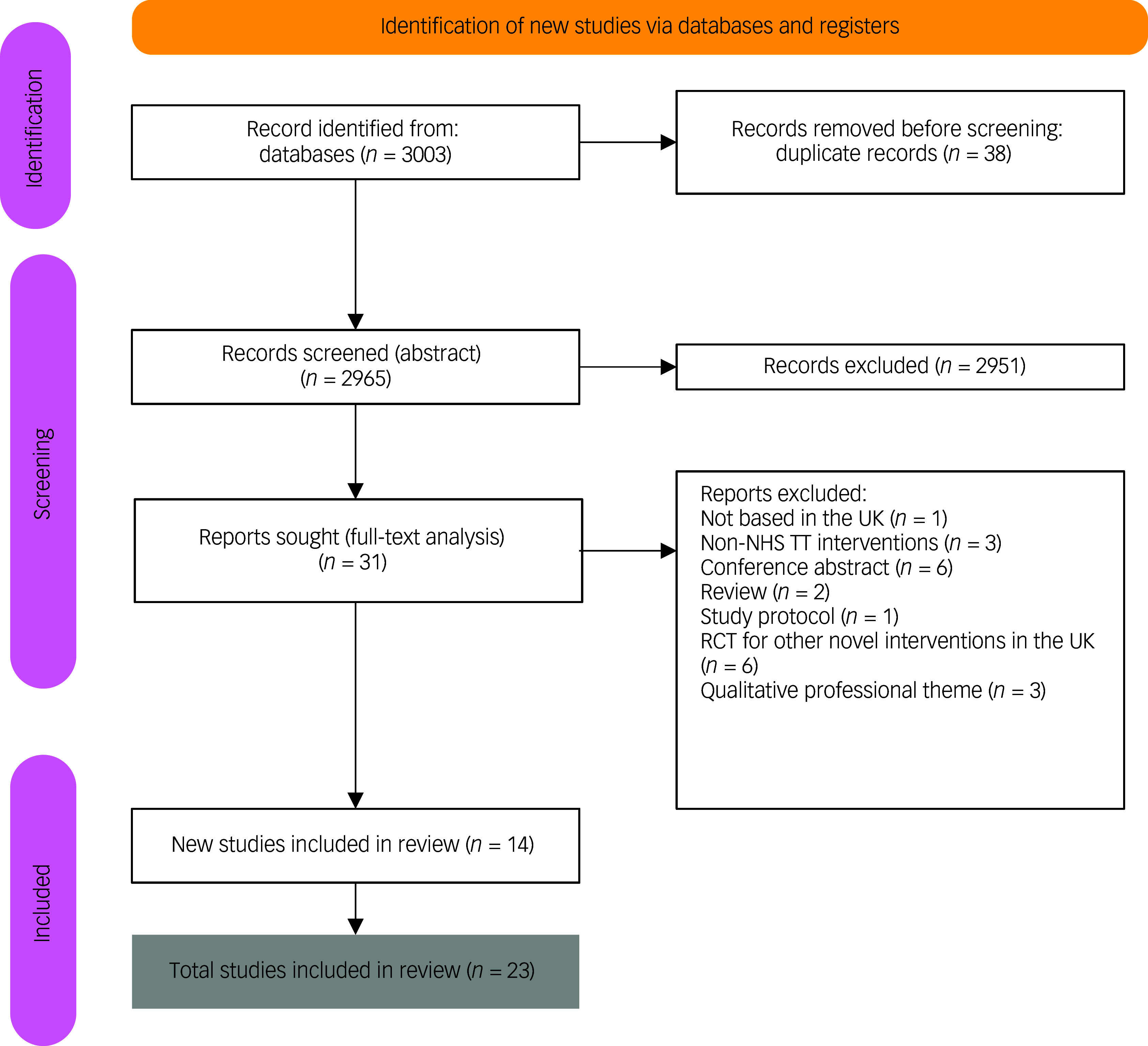
Flowchart of studies included in a systematic review on care pathways and treatment outcomes for individuals with physical long-term conditions. NHS TT, National Health Service Talking Therapies; RCT, randomised control trial.

Eligible studies were identified through database and manual searches, with titles, abstracts and full texts independently screened by two reviewers (M.C.A., J.H.). Discrepancies were resolved through discussion with a third reviewer (S.N.). The initial and updated searches (covering studies published from 2001 onwards) yielded 3003 records; following the removal of 38 duplicates, the remaining 2965 records underwent title and abstract screening, followed by full-text assessment of 31 articles. An additional 15 studies were identified in the updated December 2024 search. In total, 24 studies met the inclusion criteria and were included in the review (Fig. 1; PRISMA flowchart). Included were five additional papers for narrative synthesis focused on treatment accessibility, uptake or engagement but that did not report treatment outcomes. In this review, LTCs were conceptualised to include chronic physical health conditions that are commonly co-occurring with mental health conditions. In order to demonstrate NHS TT condition-specific care delivery and outcome, we included the following studies: Young et al (deaf patients^15^), Bell et al (dementia^16^), Petrochilos et al (functional neurological symptom disorders^17^) and Kenwright et al (inflammatory bowel disease^18^).

### Data collection and extraction

Data extraction was performed on 18 February 2025. Two reviewers (M.C.A., A.D.) independently extracted data using a prespecified form. Extracted information included the following: (a) study characteristics (e.g. authorship, publication year, study design, number of participating services); (b) sample characteristics (e.g. age, gender, ethnicity, sample size); (c) intervention and comparator details (e.g. NHS TT care pathway, type of therapy, number and type of LTCs); and (d) treatment effectiveness outcomes (reliable recovery, reliable improvement). Reliable Improvement is defined as the statistically significant reduction in symptom scores between assessment and treatment end that exceeds the measure’s reliable change index (≥6 points on PHQ-9 or ≥4 points on GAD-7).^19,20^ Reliable recovery indicates the combined outcomes of both reliable improvement and final score below the clinical cut-off threshold (PHQ-9 <10 points and GAD-7 <8 points), signifying remission from clinical ‘caseness’. Conversely, reliable deterioration denotes an increase in symptom scores beyond the reliable change index^21,22^ and service use, and in other variables (e.g. predictors of service uptake, engagement and outcomes). Where key information (e.g. gender distribution, ethnicity, LTC type) was missing, we contacted the study authors. This review includes studies that reported depression and anxiety outcomes using PHQ-9 and GAD-7. These measures are mandated across all NHS TT services in England and form the basis of the national minimum data-set for the assessment of treatment progress and determination of reliable improvement, recovery and deterioration.^23^ While other validated instruments (e.g. the Beck Depression and Anxiety Inventories) are psychometrically sound, they are not routinely implemented within NHS TT and are rarely reported in service-level evaluation. Restricting inclusion to studies using PHQ-9 and GAD-7, therefore, ensured consistency in outcome measurements, comparability across studies and alignment with national reporting standards.

### Quality rating

The Newcastle–Ottawa Scale (NOS) was used to assess study quality across three domains: selection (up to four points), comparability (up to two points) and outcome measurement (up to three points). We also assessed the clarity and robustness of LTC definitions. Each study was independently scored by two reviewers (M.C.A., A.D.), with disagreements resolved through discussion with a third reviewer (J.H.). Based on total scores, studies were categorised into low quality (0–2 points), fair quality (3–5 points) or high quality (6–9 points). All studies were included in the review, regardless of their quality rating; however, sensitivity analyses were conducted to assess the potential impact of lower-quality studies on the findings.

### Data synthesis and analysis

A narrative synthesis was conducted following the Economic and Social Research Council recommendations, including the following:

- development of a preliminary synthesis;
- exploration of relationships within and between studies;
- assessment of the robustness of studies.

Descriptive statistics summarised the study characteristics and outcomes. For dichotomous outcomes (e.g. reliable recovery, reliable improvement), we extracted odds ratios and 95% confidence intervals. For continuous outcome measures (e.g. GAD-7, PHQ-9), we used standardised mean differences and 95% confidence intervals, focusing on change from baseline. Categorical outcomes (e.g. reliable recovery, reliable improvement, deterioration) were analysed as odds ratios with 95% confidence intervals. Heterogeneity was assessed using the *I* ^2^ statistic, with the following recommended thresholds:^24^ 0–40%, minimal; 30–60%, moderate; 50–90%, substantial; and 75–100%, considerable heterogeneity. Meta-analyses were conducted using random-effects models due to methodological and clinical heterogeneity. Given the limited number of studies and variables reported, subgroup analyses were not possible. Where available, we compared planned versus reported outcomes to assess selective reporting bias. We also conducted a sensitivity analysis excluding low-quality studies. Forest, funnel and bias plots were generated using the ‘met’ package. Effect sizes were calculated from available data where not directly reported. Statistical analyses were performed on R, version 4.5.0 for macOS and Windows (R Foundation for Statistical Computing, Vienna, Austria; https://www.r-project.org/) and R Studio, version 2025.09.1+401 (Posit Software, Boston, Massachusetts, USA; https://posit.co/download/rstudio-desktop/).

## Results

A total of 3003 records were retrieved from the database searches; following removal of 38 duplicates, 2965 titles and abstracts were screened. Of these, 31 full-text articles were assessed, with 9 studies met the inclusion criteria in the initial search (November 2022). The updated search (December 2024) identified an additional 14 studies. Overall, 23 studies were included in the final review. The PRISMA flow diagram summarising the selection process is presented in Fig. 1.

### Study characteristics

The characteristics of the included studies are summarised in Supplementary Table 1. Twenty studies employed cohort design, while three were follow-up analyses of RCTs.^17,25,26^ Sample sizes ranged from 52^25^ to 2 515 402,^27^ with mean age ranging from 38 to 64 years. Most studies included a higher proportion of female participants (54–69%) and those of predominantly White ethnicity (65–92%). The proportion of individuals with LTCs varied from 4 to 100%.

### NHS TT-LTC implementation: pre- versus post-2018

Of the included studies, 10 (42%) were published before the 2018 implementation of the NHS TT-LTC integrative care pathway. Among these, three evaluated LTC as a primary predictor,^25,28,29^ one included LTC status as a covariate^30^ and three focused on specific LTCs (e.g. diabetes, hearing impairment, irritable bowel syndrome^15,18,28^). The remaining 14 studies (58%) were conducted post-implementation. Five explicitly evaluated LTCs as primary predictors of treatment outcomes,^31–35^ four included LTC status as an adjustment variable^36–39^ and four examined specific LTCs (e.g. dementia, neurological disorders, psychosis, autism^16,17,27,33^). Despite the policy emphasis on integration, post-2018 evaluations remain limited and heterogeneous in both design and focus.

### Outcome measures

Approximately half of the studies used NHS TT national outcome benchmarks, including reliable recovery, reliable improvement and reliable deterioration. Four studies reported general recovery rather than reliable recovery.^25,33,37,40^ Fourteen studies used continuous measures (e.g. PHQ-9, GAD-7), while five examined treatment uptake and engagement.^34,38,41,42,43^

### Study quality

Quality assessment using NOS found 15 studies (62.5%) to be of high quality (score ≥6), while the remaining 9 studies (37.5%) were of fair or low quality. High-quality studies typically reported robust sampling, community-based recruitment and the use of electronic health records.^29,41,42^ Methodological limitations in lower-quality studies^15,25,28^ included unclear sampling, limited data transparency and inconsistent LTC definitions. Although no consistent pattern of increasing study quality was observed over time, more recent publications (from 2018 onwards) generally demonstrated stronger methodological rigour, larger sample size and improved reporting of treatment outcomes and subgroup analyses, reflecting maturation of the NHS TT data infrastructure.

### Methodology challenges

Methodological heterogeneity was substantial. Most studies had used logistic regression for binary outcomes or linear regression for continuous outcomes.^27,31,43^ Seven studies relied solely on descriptive statistics, and two employed machine learning techniques.^18,28^ There was also considerable variation in model specifications, participant demographics, LTC definitions, follow-up duration and treatment modalities, limiting direct comparisons.

### Service uptake and engagement evaluation

**Fig. 2:**
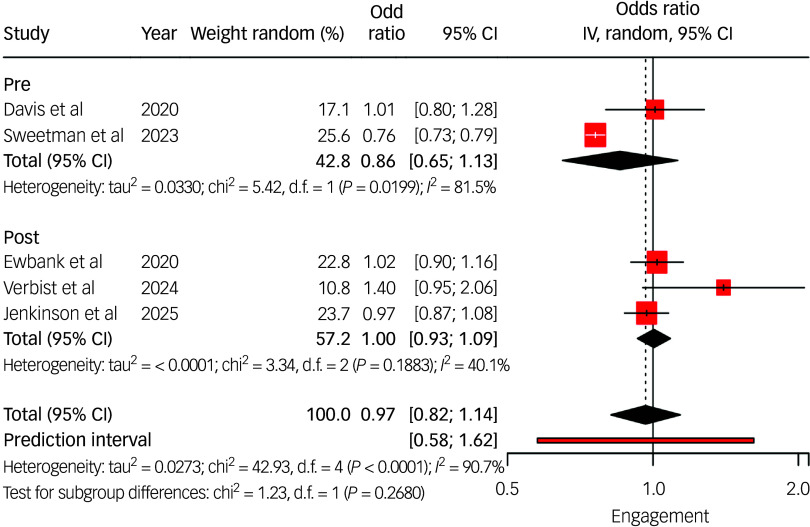
Long-term condition (LTC) engagement odds ratio instrumental variable (IV) meta-analysis. IV estimates causal effect of having an LTC on the outcome by combining study estimates with intervention-induced variation. Studies listed include the author(s) and year of publication; pre/post indicates whether the data collection period was conducted pre- or post-National Health Service Talking Therapies-LTC.

Pooled analysis (Fig. 2) showed that individuals with LTCs had similar odds of engaging in treatment compared with those without (odds ratios ranged from 1.01 to 1.40). Two studies^34,43^ found no differences in initial assessment attendance by LTC status. By contrast, Sweetman et al^38^ reported significantly lower engagement among adults with long-term sickness or disability (adjusted odds ratio 0.76, 95% CI: 0.73–0.79).

### Therapy type and medium, duration and numbers of sessions

Nine studies reported CBT as their primary intervention^17,25,28,33,35–37,39,42^ and four on counselling.^28,36,39,40^ One study reported the use of acceptance and commitment therapy.^28^ Kellett et al^28^ reported using pacing and motivational interviewing; four studies reported on the use of digital interventions.^32,35,42,43^

Regarding the media used for those with LTC, two studies^18,37^ specified the use of face-to-face intervention. Kenwright et al^18^ and Boyd et al^37^ referred to the use of telephones, while four studies used digital media.^32,35,42,43^ None of the studies reported on hybrid face-to-face and online intervention.

Seventeen studies reported on the duration and number of sessions;^16–18,25–29,32–37,39,40,42^ the number of sessions between these studies ranged from 2 to 16. Delgadillo et al,^29^ Wroe et al,^26^ Ewbank et al^42^ and Bell et al^16^ reported observing a dose–response relationship between duration and number of sessions with reliable improvement and recovery; Wroe et al^26^ and Bell et al^16^ reported this outcome specifically for those with an LTC. Wroe et al^26^ reported a significant main effect of time from pre- to post-intervention, further confirming that a longer time interval is associated with improvement in post-treatment outcome scores. Bell et al^16^ noted that more therapy sessions were associated with better treatment outcomes (recovery: odds ratio 1.12, CI: 1.09–1.16, *P* < 0.001) for those with dementia.

### Treatment outcomes in individuals with LTCs

**Fig. 3:**
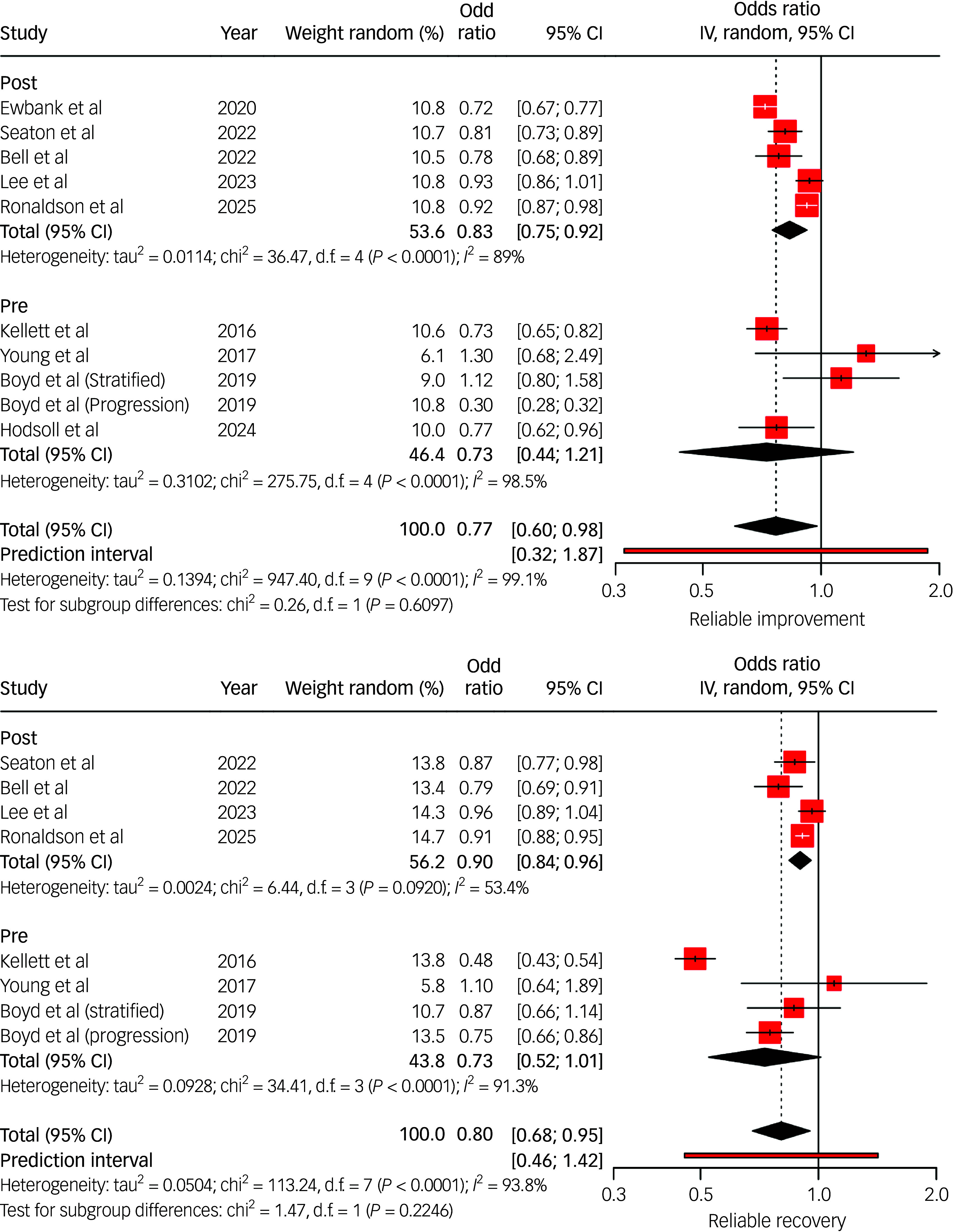
Random effects (instrumental variable (IV)) meta-analysis of reliable improvement (top) and reliable recovery (bottom) odds ratios for individuals with long-term conditions (LTCs) accessing National Health Service Talking Therapies. Studies listed include the author(s) and year of publication; pre/post indicates whether the data collection period was conducted pre- or post-National Health Service Talking Therapies-LTC. In the case of Boyd et al, odds ratios were extracted from their data for both stratified and progressive step-care models. IV estimates causal effect of having an LTC on the outcome by combining study estimates with intervention-induced variation.

**Fig. 4:**
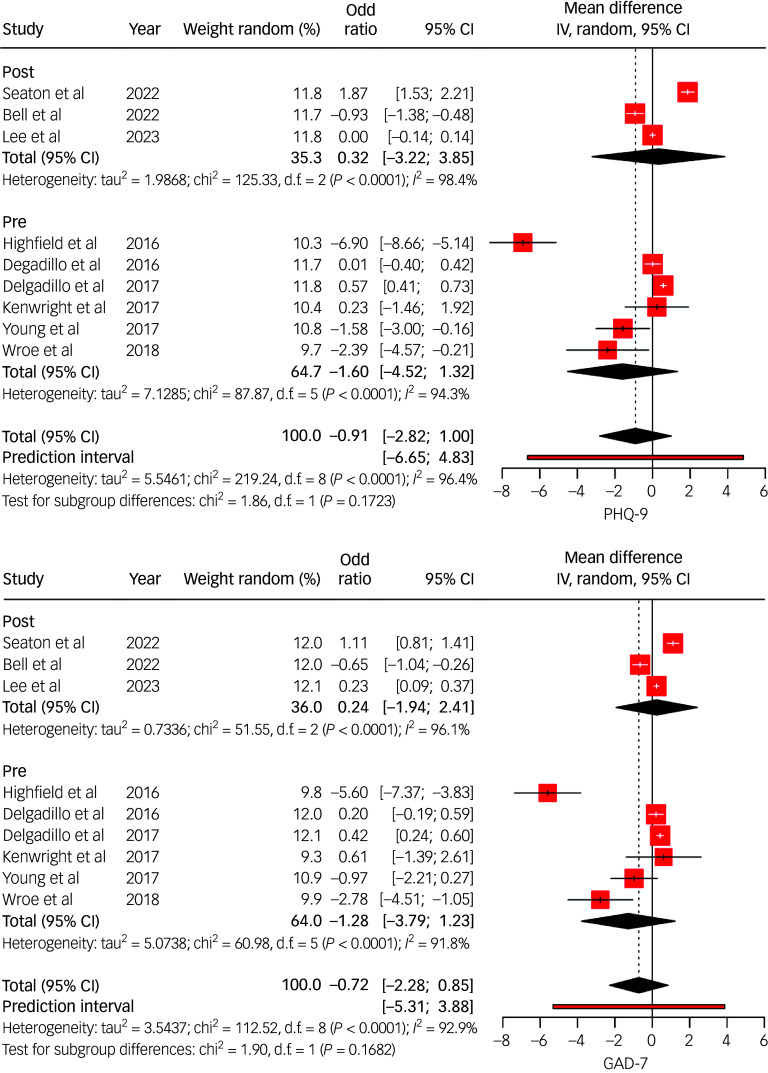
Random effects (instrumental variable (IV)) meta-analysis of Patient Health Questionnaire 9 (PHQ-9) (top) and General Anxiety Disorder 7 (GAD-7) (bottom) mean differences for individuals with long-term conditions (LTCs) accessing National Health Service Talking Therapies (NHS TT). Studies listed include the author(s) and year of publication; pre/post indicates whether the data collection period was conducted pre- or post-NHS TT-LTC. IV estimates causal effect of having an LTC on the outcome by combining study estimates with intervention-induced variation.

Thirteen studies compared outcomes between individuals with LTCs and those without, using categorical and continuous metrics (Figs 3 and 4). Pooled results indicated that individuals with LTCs had significantly poorer treatment outcomes, characterised by higher post-treatment psychological distress (Fig. 4) and lower rates of reliable recovery and improvement (recovery odds ratio range 0.48–0.96; improvement odds ratio range 0.30–0.93; Fig. 3). Subgroup analysis from Seaton et al^31^ found no moderating effects of demographic variables on outcomes, suggesting broadly consistent disparities. Petrochilos et al^17^ reported that, although their findings were specific to functional neurological symptom disorder, CBT treatment maintained improvement 6 months post-treatment.

### Effectiveness of LTC-tailored interventions

Three RCTs evaluated LTC-specific interventions (Fig. 4). All reported more favourable outcomes compared with standard care, particularly when delivered at high intensity or by LTC-trained clinicians. For example, Highfield et al^25^ reported that patients receiving tailored step 3 interventions had 3.5- and 8.6-fold higher odds of recovery from depression and anxiety, respectively, compared with step 2 treatment as usual.

### Predictors of treatment outcomes

Having an LTC was consistently associated with poorer outcomes. Other key predictors included employment status, deprivation, gender, ethnicity, antidepressant use and baseline severity. For example, Delgadillo et al^29^ found significantly worse post-treatment scores among South Asian and dual-heritage patients. Bell et al^16^ identified medication use, functional impairment and age as important modifiers in dementia care pathways.

Only four studies evaluated stepped-care models, with Boyd et al^37^ reporting better recovery outcomes in progressive models compared with stratified approaches. Reporting on care pathways remained limited and inconsistent across studies.

## Discussion

This systematic review and meta-analysis synthesised evidence on the effectiveness of NHS TT for adults with coexisting LTCs. Across two observational and interventional studies, consistent evidence emerged of poorer treatment outcomes for individuals with LTCs relative to those without, with lower rates of reliable recovery and improvement and higher residual symptoms of depression and anxiety. These patterns suggest that standardised, non-tailored interventions may not sufficiently address the complexity of co-occurring physical and psychological needs in LTC populations. Importantly, these associations persisted independently of demographic characteristics including age, gender and ethnicity (Seaton et al^31,35^), indicating an intrinsic link between LTC status and reduced therapeutic benefit within the NHS TT service.

Importantly, evidence from three RCTs supported the potential of tailored, high-intensity interventions to improve outcomes for individuals with LTCs. For example, Highfield et al^25^ found that LTC-specific step 3 interventions, delivered by trained practitioners, significantly outperformed standard step 2 interventions, resulting in a 4-fold increase in reliable recovery rates for depression. This finding highlights how both the depth of clinical input and the relevance of practitioner knowledge to the patient’s physical health context can significantly influence treatment success. In practice, this means that standard care pathways may fall short for individuals with LTCs, and that targeted upskilling of practitioners in LTC-informed psychological care should be prioritised to improve mental health outcomes in this high-risk group. However, no studies have evaluated the recent integration of NHS TT and LTCs.

LTC status, however, did not appear to be a universal barrier to accessing NHS TT services or initiating treatment. Most studies reported comparable engagement rates between patients with and without physical LTCs. However, heterogeneity was evident: Sweetman et al,^38^ for instance, identified lower treatment uptake among individuals reporting long-term disability (adjusted odds ratio 0.76), highlighting that the functional impact or severity of LTCs may influence engagement. These findings suggest that service access should be evaluated not only by diagnostic presence but also by the functional consequences of chronic illness. For instance, while many adults with LTCs may meet diagnostic thresholds for depression or anxiety, those experiencing more severe impairments (e.g. mobility limitations, chronic pain or fatigue) may face greater barriers to engaging in treatment. These functional difficulties can affect their ability to attend appointments, complete therapeutic tasks or sustain engagement, regardless of the underlying mental health diagnosis. As such, assessment of service accessibility should consider how physical disorder impacts day-to-day functioning, and services should adapt to accommodate these challenges (e.g. via flexible scheduling, remote delivery options or integrated physical and psychological support).

The review also highlighted several additional predictors of poorer outcomes in LTC populations, including unemployment, socioeconomic deprivation, ethnic minority background and psychotropic medication use. Delgadillo et al^29^ reported higher post-treatment distress among South Asian and dual-heritage groups, and Bell et al^16^ identified antidepressant use and older age as negative prognostic factors in people with dementia. These findings point to the combined effect of multimorbidity and social disadvantage, reinforcing the need for a biopsychosocial approach to treatment planning. Furthermore, evidence from the above-mentioned studies suggests a dose–response relationship between duration and number of sessions with reliable improvement and recovery for those with LTCs.

Evidence regarding the organisation of care was limited. Only four studies explored stepped or stratified models of delivery. Among these, Boyd et al^37^ reported better recovery outcomes from progressive models tailored to disability, supporting the case for care pathways that incorporate patient complexity and functionality rather than relying solely on symptom thresholds. Although therapy type and medium were reported across studies, their specific effects for individuals with LTCs were largely under-explored. Interestingly, there is limited literature on the use of digital or online interventions for those with LTCs, and none reporting on the use of mobile mental health apps as a form of therapy. A 2021 systematic review by Eisenstadt et al identified small to medium positive effects on mental health symptoms, well-being and emotional regulation.^44^ However, among those with LTCs, Seaton et al^35^ reported suboptimal outcomes when utilising LTC-specific digital interventions. Nonetheless, given that this is a single study, the effectiveness of digital interventions for people with LTCs remains inconclusive.

A critical implication of this review concerns the suitability of current outcome measures for LTC populations. While both PHQ-9 and GAD-7 remain core components of NHS TT service monitoring, their sensitivity to change in individuals with LTCs might be limited. Elevated scores in this group may reflect persistent illness-related distress rather than modifiable psychiatric symptoms, resulting in an underestimation of therapeutic benefits. This raises the possibility that some of the observed poorer outcomes may be artefactual. Future evaluations should incorporate more nuanced outcome measures – such as functioning, illness-related distress and quality of life – that better reflect clinically meaningful change in LTC populations.

Although individuals with long-term physical health conditions engage with NHS TT services at rates comparable to those without LTCs, they experience consistently poorer outcomes under standard care models. Tailored interventions, especially those delivered by trained practitioners in high-intensity settings, appear to offer a promising route to address this disparity. Going forward, it is important to embed LTC-sensitive approaches into service design, including outcome monitoring and practitioner training, to enhance mental health support for this complex and growing patient group.

### Strengths and limitations

This review followed PRISMA guidelines and included a comprehensive, up-to-date search strategy. By using standardised effect estimates (odds ratios), we were able to summarise treatment differences across heterogeneous designs. However, several limitations should be acknowledged. There was marked variability in how LTCs were defined and operationalised, and few studies captured LTC severity, co-occurrence or chronicity. Adverse effects were not the primary focus of this review, and none of the included studies reported these explicitly. This lack of systematic assessment of potential harms represents a vital evidence gap within the NHS TT that should be addressed in future evaluations of the service. Reporting of care models and delivery pathways was sparse, precluding robust inferences about optimal implementation. Many included studies lacked ethnic diversity and quality was mixed, with over a third of included studies judged to be of low quality. Finally, publication bias was not formally assessed, but it is probably present given the absence of unpublished or null results. Information on the mode of delivery (face-to-face, online or hybrid) and the use of digital web-based programmes was provided, but only one study evaluated their effectiveness for those with LTCs. None of the studies involved the use of apps as a form of therapy for those with LTCs. As a result, we were unable to assess the potential impact of these delivery modalities, highlighting an important area for future research. Furthermore, findings regarding associations that appear independent of demographic characteristics should be interpreted with caution, because these were derived from a single study and may not fully account for unmeasured or residual confounding.

### Policy and practice implications

The findings highlight several actionable priorities. First, systematic recording of LTCs within NHS TT services is essential to inform care planning and equity monitoring. Second, there is a clear case for scaling up tailored interventions and training practitioners in managing mental–physical health multimorbidity. Third, while integrated care pathways show promise, their structure and implementation remain highly variable; future work should identify scalable, sustainable models that deliver improved outcomes across clinical settings. Finally, addressing social determinants, such as unemployment, deprivation and minoritised ethnic backgrounds, will be key to reducing disparities and supporting recovery in under-served populations.

## Supporting information

Abichi et al. supplementary material Abichi et al. supplementary material

## Data Availability

All data underlying the conclusions of this review are included in the published article and its supplementary materials. No new data-sets were generated in the course of this study; all analyses are based on previously published work cited within the manuscript.

## Supplementary material

The supplementary material is available online at https://doi.org/10.1192/bjo.2025.10957
