# Supplementary material for: Current treatment outcomes and care pathways for people with comorbid physical and mental health conditions using NHS Talking Therapies services in the UK: systematic review of quantitative studies
Source: BJPsych Open. 2026 Feb 5;12(2):e56. doi: 10.1192/bjo.2025.10957 (PMC12926884; doi:10.1192/bjo.2025.10957)
Supplement: Abichi et al. supplementary material [file S2056472425109575sup001.docx]

| TABLE 1: STUDY CHARACTERISTICS | | | | | | | | | | | | | | | | | | | | | | | | | |
| --- | --- | --- | --- | --- | --- | --- | --- | --- | --- | --- | --- | --- | --- | --- | --- | --- | --- | --- | --- | --- | --- | --- | --- | --- | --- |
| **PRE-NHS TT- LTC IMPLEMENTATION** | | | | | | | | | | | | | | | | | | | | | | | | | |
| **Author** | **Year** | | **Study Design** | | | **Service** | **Analysed** | | | **Long-Term Condition** | **Analysed Measure** | | **Intervention (Step of Care)** | | | **Average Number of Session** | **Outcome Measure** | | | **Findings post-treatment** | **Predictors of Outcome** | **Paper Quality** | **Methodology** |  |  |
| (25) Highfield et al, 2016 | 2014 | | Randomised Controlled Trial | | | West Midlands | N=28 Adapted Step 2 Step 3 N=52 Non-adapted S3 N=24 Adapted LTC S3 N=28 | | | All LTCs with Depression and Anxiety | Treatment Outcomes | | “Mind & Body” CBT  (Step 2  Step 3) | | | 7 | PHQ9  GAD7 Recovery | | | Step 2: PHQ9 *_Mdiff_* =-6.9, CI = -5.72 - -8.08 p=0.01 GAD 7 *_Mdiff_* = -5.3 CI = -4.03 - -6.57 p=0.05 Step 3:Adapted;  PHQ9 recovery = 58%, GAD7 = 54% Adapted with trained and supervised workers;  PHQ9 recovery = 79% and GAD7= 90% | Tailored Treatment Trained Therapist for LTC | 3 | Description Analysis: Means and Proportions |  |  |
| (28)  Kellett et al., 2016 | 2016 | | Observational Cohort Study – | | | Sheffield, England | General Population N= 10,469  LTC N=844 MUS= 172 | | | LTCs with Depression and Anxiety | Treatment Outcomes | | Low Intensity (PWP) GSH  Pacing  Motivational Interview (Step2)  CBT, ACT and Counselling (Step up to 3) | | | Step 2: 1-6 (30-35min)  Step 3 : 20 | PHQ9 GAD7 Reliable Recovery Reliable Improvement Reliable Deterioration | | | 20.47% -LTC achieve Reliable Recovery 17.39% - MUS achieves Reliable Recovery 25.17% Generic NHS TT achieve Reliable Recovery No effect size reports for LTC vs Non-LTC | LTC | 5 | Percentage Proportions Chi-Squared  Partial eta squared |  |  |
| (30) Delgadillo et al., 2016 | 2016 | | Observational Cohort study | | | North of England | General population N=1,347  LTC Numbers not reported | | | The general population with Depression and anxiety, adjusted for LTC | Treatment Outcome | | Step 2 or Step 3 Step up from 2 to 3 | | | Not Reported | PHQ9  GAD7 | | | LTC (not a predictor of outcomes) PHQ9 b=0.01 SE;0.209, p=0.961 GAD7 b=0.202, SE; 0.198, p=0.307 | GAD7 Baseline PHQ9 Expectancy PHQ9 Age Employment Disabled Baseline WSAS | 5 | Multivariate logistic regression and Backward predictor elimination |  |  |
| (18) Kenwright et al., 2017 | 2017 | | Observational Cohort study | | | North Midlands, England | IBS Patients N=138 | | | Irritable Bowel Syndrome (IBS) with Bowel Control Anxiety | Treatment Outcomes | | GSH  (Step 2)  CBT Step 3 | | | Step 2: 10 Telephone  2 face to face  Step 3- Varied across patients | PHQ9  GAD7 | | | Between those with BCA and those without BCA, PHQ9 *_Mdiff_* = 0.23 GAD7 *_Mdiff_* = 0.61 | Not reported | 4 | No control group Presented and described no inferential statistics |  |  |
| (15)  Young et al 2017 | 2017 | | Observational Cohort study | | | England | General Population N=545 LTC N=22 (4%) | | | Deaf Patients with Depression and Anxiety | Treatment Outcomes | | Step 2  Step 3 | | | Not Reported | Reliable recovery | | | There is no significant difference in reliable recovery between standard NHS TT and BSL-NHS TT | Tailored Treatment | 5 | T-test Pearson’s Chi-squared Mann-Whitney U-test |  |  |
| (29) Delgadillo et al., 2017 | 2017 | | Observational Cohort Study – | | | North of England | General Pop N= 28,498  LTC Present N=6,616 (23%) | | | LTC with Depression and anxiety | Treatment Outcome Service Access | | Step 2 Step 3 | | | Not Reported | PHQ9  GAD7 | | | PHQ-9 aM_diff_ 0.57 (s.e = 0.09, p<0.001), ES_PHQ_ = 0.10GAD aM_diff_ 0.42 (s.e = 0.08, p<0.001, ES_GAD_= 0.08High Intensity Treatment LTC ORs=1.23-1.66; p<0.05) | LTC | 9 | Seemingly unrelated regression (SUR) |  |  |
| (26)  Wroe et al., 2018 | 2018 | | Randomised Controlled Trial | | | Berkshire, England | Patients with Type II Diabetes N= 115 Received diabetes-specific intervention N= 52 Received usual care NHS TT intervention N=63 | | | Type 2 Diabetes | Treatment Outcomes | | Diabetes specific Step 2 | | | >4 | PHQ9  GAD7 | | | PHQ9 Mdiff= -2.78,CI= -0.67 - -4.89, p= 0.01 GAD7 Mdiff = -2.39 CI = -0.53 - -4.25, p=0.012 | Tailored Treatment | 5 | t-test for both psychological and physiological measures such as HbA1c |  |  |
| (36)  Moller et al., 2019 | 2019 | | Observational  Cross-sectional study | | | England | N=517,942  LTC Number not reported | | | LTC with Depression and Anxiety | Treatment Outcome | | Step 3: CBT  Counselling | | | CBT: 7.4  CfD: 6.3 | Recovery | | | Speech, sight, learning disability, and physical health conditions recovery rates 42-48% | Physical Health Conditions | 4 | Descriptive |  |  |
| (41)  Davis et al., 2020 | 2020 | | Observational  Cross-sectional study | | | Not report(19 NHS TT sites) | Referrals N=129,321  LTC numbers not reported | | | LTC with Depression and anxiety | Service Access | | Not reported | | | Not reported | Engagement | | | Engagement = aOR=1.01, SE 0.12, P=0.95 | LTC | 9 | Generalised linear Mixed effects model (Logistic Regression) |  |  |
| (42) Ewbank et al., 2020 | 2020 | | Observational Cohort study | | | UK | General Population N= 17,525  LTC number not reported | | | The general population with Depression and anxiety, adjusted for LTC | Treatment Outcome  Service Access | | Step 2 - Internet enabled CBT | | | 6.2 (2.9) | Reliable improvement - 63.4% Engagement - 87.3% | | | Engagement - 1.02(0.90-1.15) p=0.81 Reliable improvement - 0.72 (0.66-0.80) p<0.001 | LTC | 6 | Deep Learning |  |  |
| (38) Sweetman et al., 2023 | 2023 | | Observational Cohort study | | | North of England | General Population N=97,020  LTC number not reported | | | The general population with Depression and anxiety adjusted for Long-term sick/disabled | Service Access | | Not reported | | | Not reported | Uptake  Engagement | | | Long-term sick/disabled (Employment Status) Uptake; 0.73 (0.69-0.78) P<0.001  Engagement; 0.76 (0.72-0.81) P<0.001 | Long-term sick/disabled | 7 | Logistic Regression |  |  |
| (40)  Hodsoll et al., 2024 | 2024 | | Observational Cohort study | | | London | N=263 | | | The general population with Depression and anxiety, adjusted for CIRS (Physical Health) | Treatment Outcome | | CBT/GSH  (Step 2)  CBT/ Counselling (Step 3) | | | 8 | PHQ9 GAD7 Recovery Reliable Improvement | | | Chronic Inflammatory Response Syndrome (CIRS) Recovery 0.83 (0.61-1.12) p=0.219 Reliable Improvement 0.77 (0.58-1.02) p=0.064 | CIRS insignificant | 6 | Bayesian Projective predictions for variable selection Leave-one-cross-validation |  |  |
| **POST NHS TT- LTC IMPLEMENTATION** | | | | | | | | | | | | | | | | | | | | | | | | | |
| **Author** | | **Year** | | **Study Design** | **Service** | | | **Analysed** | **Long-Term Condition** | | | **Analysed Measure** | | **Intervention (Step of Care)** | **Average Number of Session** | | | **Outcome Measure** | **Findings post-treatment** | | **Predictors of Outcome** | **Paper Quality** | **Methodology** | |  |
| (37)  Boyd et al, 2019 | | 2019 | | Observational Cohort study | North East England | | | N=16,723 Disabilities N= 1,276 (8%) | Disability with Depression and Anxiety | | | Treatment Outcome | | Step 2: Telephone, GSH, Psychoeducation  Face to faceGSH  Step 3 Step up from 2 to 3 | Step2:  4  6-8: face to face GSH  Step 3:12-16 | | | Recovery | Recovery aOR = 0.975, CI=0.7878-1.209, p=0.818 | | Stepped Care | 9 | Logistic Regression | |  |
| (39)  Delgadillo & Duhne, 2020 | | 2020 | | Observational  Cohort study | North of England | | | General Population N=1,435  LTC number not reported | The general population with Depression and Anxiety LTC | | | Service Access | | CBT  CfD  Step 2 or Step 3 Step up from 2 to 3 | CBT:9.56  CfD:8.31 | | | CBT and Counselling for Depression Prognostic modelling | Those assigned to their optimal treatment were 2.33 times more likely to attain reliable and clinically significant improvement Comorbid LTC showed no difference in response to CBT or CfD | | - | 9 | Machine Learning - elastic nets with optimal scaling, decision trees | |  |
| (17)  Petrochilos et al., 2020 | | 2020 | | Randomised Control Trial with 6-month follow-up | London | | | Function Neurological Symptom disorder (FNSD) N=78 completed both the pre-program and the six-monthly review | Functional Neurological symptom disorder. (FNSD) with Depression and anxiety | | | Treatment Outcome | | CBT - Step 3 - Tailored for FNSD | 9 | | | PHQ9  GAD7 | Between Discharge and admission, significant change Between 6-months review and admission, a significant difference No significant change. Between Discharge and 6-month follow-up | | Collaborative Intervention | 4 | Friedman ANOVA Dunn Bonferroni test and effect size Kendall's W. | |  |
| (31)  Seaton et al., 2022 | | 2022 | | Observational  Cohort study | London | | | LTC N=2,075 (31%) | LTC with Depression and Anxiety | | | Treatment Outcome | | Step 2 Step 3 | Not reported | | | PHQ-ADS WSAS PHQ9 GAD7 Reliable Recovery Reliable Improvement | Recovery aOR = 0.857, CI=0.75-0.98, p=0.025 Reliable Improvement aOR = 0.807, CI=0.71-0.91, p<0.001 PHQ9 Mdiff 1.86, CI= 1.5-2.2, p<0.001  GAD7 Mdiff = 1.11, CI = 0.8-1.4P<0.001 | | LTC | 8 | Logistic Regression Linear Regression | |  |
| (16)  Bell et al., 2022 | | 2022 | | Observational Cohort study | England, UK | | | N=211 | People living with Dementia (PLWD) with Depression and anxiety (also adjusted for LTC) | | | Treatment Outcome | | Step 2 Step 3 | 5.53 (3.98) | | | PHQ9 GAD7 Reliable Recovery Reliable Improvement Reliable Deterioration | PS match (adjusted OR) Reliable Improvement 0.78 (0.66-0.93)p=0.004 Reliable Recovery 0.79 (0.66-0.94)p=0.006 Reliable Deterioration 1.31 (0.99-1.67) p=0.062 PHQ9 b=0.93 (se0.23) p<0.001 GAD7 b=0.65 (se;0.20) p=0.001 | | Dementia | 8 | Logistic Regression | |  |
| (27)  El Baou et al., 2023 | | 2023 | | Observational Cohort study | England UK | | | N = 2,515,402 GP N=8,761 Autism(0.3%) | Autism co-morbid LTC with Depression and Anxiety | | | Treatment Outcome | | Step 2 Step 3 | 6.5 (4.6) | | | PHQ9 GAD7 Reliable Recovery Reliable Improvement Reliable Deterioration | Interaction analysis LTC/no LTC and Autism/control PSM cohort: No significant interaction for Reliable Improvement, Reliable Recovery, and Reliable Deterioration. Full Sample Cohort: There is an interactive effect for Reliable Recovery and Reliable Deterioration | | Autism | 7 | Propensity Score Matching Logistic Regression Interaction analysis | |  |
| (35)  Seaton et al., 2023 | | 2023 | | Observation Cohort Study 2019 | England | | | LTC N=76 | LTC with depression and anxiety | | | Treatment Outcome | | LTC Digital COMPASS Step 2 | 11 | | | PHQ9  GAD7  PHQ-ADS | Pre and Post-intervention  PHQ9 Mdiff = -2.76, cohen d=  -0.38)  GAD7 Mdiff = -2.30 cohen d=  -0.42  PHQ9-ADS Mdiff = -4.87 cohen d = -0.42 | | LTC | 3 | Paired t-test | |  |
| (32)  Lee et al., 2023 | | 2024 | | Observational Cohort study | Berkshire Healthcare, NHS | | | N=21,501 LTC N=4,024 (19%) | LTC with Depression and Anxiety | | | Treatment Outcome | | Step 2:  iCBT  GSH  PGT | 5.15 (2.64) | | | PHQ9  GAD7 | PHQ9 Mdiff = 0.03 GAD7 Mdiff = 0.24 WSAS Mdiff = 0.04 | | LTC Intervention | 7 | Analysis of Covariance (ANCOVA) | |  |
| (34)  Verbist et al., 2024 | | 2024 | | Observational Cohort study | England | | | General population N=13,019  *LTC =*  *Pre-lockdown N= 535 (18.4%)*  *During lockdown N= 634 (19.5%)*  *Post lockdown N=874 (24.8%)* | LTC with Depression and Anxiety | | | Service Access | | Not reported | 2 (range 2-19) | | | Engagement | Significantly Higher engagement for individuals with LTC during lockdown (planned discharged) Chi^2^ = (1,1552)=5.89, p=0.01Accessing treatment is insignificant LTC p=0.91LTC is 1.4 times more likely to engage with treatment | | LTC | 6 | Chi-Square Multiple Logistic Regression | |  |
| (43)  Jenkinson et al., 2025 | | 2025 | | Observational Cohort study | London | | | N=17,095 | LTC with Depression and Anxiety | | | Service Access | | Step 2 | Not reported | | | Uptake Engagement | Attended Assessment aOR = 1.16, CI 0.99-1.35, p=0.062 Engagement aOR = 0.97, CI= 0.86-1.07, p=0.563 Received Internet-enabled therapy aOR=0.74, CI = 0.60-0.90, P=0.003 | | LTC | 8 | Logistic Regression | |  |
| (33)  Ronaldson et al., 2025 | | 2025 | | Observational Cohort study | London | | | N=35,814 | Multiple LTC with Depression and Anxiety | | | Treatment Outcome | | Step 2:  GSH,  iCBT,  Psychoeducation,  BA  Step3:  Face to Face CBT,  IPT,  BA,  Counselling,  Psychodynamic therapy | 8 (range 5-12) | | | Recovery, Reliable Improvement | Recovery: aOR-0.91(0.88-0.95) Reliable Improvement: 0.92(0.86-0.96) | | MLTC LTC | 8 | Regression | |  |
| LTC = Long Term Condition, MLTC = Multiple Long Term Conditions, MUS= Medically unexplained Symptoms, IAPT= Improving Access to Psychological Therapies (now known as NHS Talking Therapies), CIRS= Chronic Inflammatory Response Syndrome, PLWD= People living with Dementia, FNSD = Functional Neurological Symptom Disorder, Step 2 = Low-intensity Therapy, Step 3 = High-Intensity Therapy, WSAS =Work and Social Adjustment Scale, PHQ9= Patient Health Questionnaire 9 (Depression), GAD7 = Generalised Anxiety Disorder 7 (Anxiety), CHR-P= Clinical High-Risk Psychosis, NHS= National Health Service, CCG= Clinical Commissioning Group, OR= Odd Ratio, aOR= Adjusted Odds Ratio, MANCOVA= Multivariate analysis of Covariance, ANCOVA = Analysis of covariance, ANOVA = Analysis of Variance, GSH- Guided Selp Help, BA- Behavioural Activaiton, CBT | | | | | | | | | | | | | | | | | | | | | | | | | |
